# Co-infection of COVID-19 and parasitic diseases: A systematic review

**DOI:** 10.1016/j.parepi.2023.e00299

**Published:** 2023-03-30

**Authors:** Fatemeh Nemati Zargaran, Mosayeb Rostamian, Sara Kooti, Hamid Madanchi, Keyghobad Ghadiri

**Affiliations:** a Infectious Diseases Research Center, Health Institute, Kermanshah University of Medical Sciences, Kermanshah, Iran; b Behbahan Faculty of Medical Sciences, Behbahan, Iran; c Department of Medical Biotechnology, School of Medicine, Semnan University of Medical Sciences, Semnan, Iran; d Drug Design and Bioinformatics Unit, Department of Medical Biotechnology, Biotechnology Research Center, Pasteur Institute of Iran, Tehran, Iran

**Keywords:** Co-infection, COVID-19, Parasite, Parasitic disease, Systematic review

## Abstract

Co-infection of COVID-19 with other diseases increases the challenges related to its treatment management. COVID-19 co-infection with parasites is studied with low frequency. Here, we systematically reviewed the cases of parasitic disease co-infection with COVID-19. All articles on COVID-19 co-infected with parasites (protozoa, helminths, and ectoparasites), were screened through defined inclusion/exclusion criteria.

Of 2190 records, 35 studies remained for data extraction. The majority of studies were about COVID-19 co-infected with malaria, followed by strongyloidiasis, amoebiasis, chagas, filariasis, giardiasis, leishmaniasis, lophomoniasis, myiasis, and toxoplasmosis. No or low manifestation differences were reported between the co-infected cases and naïve COVID-19 or naïve parasitic disease.

Although there was a relatively low number of reports on parasitic diseases-COVID-19 co-infection, COVID-19 and some parasitic diseases have overlapping symptoms and also COVID-19 conditions and treatment regimens may cause some parasites re-emergence, relapse, or re-activation. Therefore, more attention should be paid to the on-time diagnosis of COVID-19 and the co-infected parasites.

## 1 Introduction

COVID-9, a disease caused by the SARS-CoV-2 virus, is a worldwide pandemic that causes severe respiratory sickness and death (Huang et al., 2020). This epidemic has brought significant public health and clinical problems to humankind (Mahase, 2020). The disease is mostly spread via virus-contained droplets. The upper respiratory tract of infected cases is the first place for the virus accumulation, where goblet and ciliated cells were attacked. Similar to SARS-CoV, to start its infectious lifestyle, SARS-CoV-2 binds to the cell surface Angiotensin-converting enzyme 2 (ACE2) receptor. SARS-CoV-2-infected individuals may show mild to severe or be asymptomatic (Paces et al., 2020).

Parasitic diseases are caused by protozoa, helminths, and ectoparasites in many parts of the world in particular in the endemic regions of low-income and low-middle-income countries (LMIC). The parasites pose a significant burden on malnourished people living in unclean living circumstances (Miguel et al., 2021). Protozoan infections are a serious public health problem that mainly causes some neglected tropical diseases (NTDs) with high morbidity and mortality across the world (Hotez and Lo, 2020). Helminths are parasitic worms that cause numerous human infectious in LMIC countries (Hotez et al., 2008). Ectoparasites reside on the exterior of their hosts and the majorities are indigenous in some LMIC countries but further infestations might be linked to tourism (Heukelbach and Feldmeier, 2004).

Co-infection of COVID-19 with other diseases and infections increases the challenges related to its treatment management. There have been relatively few studies about SARS-CoV-2 co-infection with other pathogens (Zhu et al., 2020), but collecting evidence indicates that microbial co-infection increases the risk of disease severity in humans. Co-infection may raise the therapy intolerance, severely impair the host's immune system, and damage the disease's prognosis (Li and Zhou, 2013). COVID-19 disease is reported to be associated with some viral, bacterial, or fungal infections (Zhang et al., 2020), but its association with parasitic infections is not well understood.

Parasite co-infection effect on the outcome of COVID-19 is a challengeable issue and there is controversy in this regard, some agree that co-infection reduces the COVID-19 incidence rate, while others believed that parasites may weaken the effective immune responses toward protecting from COVID-19 (Bradbury et al., 2020; Cai et al., 2022; Fonte et al., 2020; Głuchowska et al., 2021). Parasite co-infection may inhibit the effective immune response to SARS-CoV-2 in the early stages of infection; thereby increase morbidity and mortality of COVID-19. It can also suppress the immune responses and mitigate SARSCoV-2 vaccine efficacy (Abdoli, 2020). Co-infection with certain organisms may also make proper illness identification difficult (Gutman et al., 2020). Moreover, regarding the fact that many people infected with SARS-CoV-2 receive immunosuppressive drugs, it is a possible risk factor for severe parasitic infections (Gautam et al., 2021).

Despite some attempts to assess the relationship between COVID-19 and parasitic diseases, the types of co-infected pathogens and the proportion of co-infection in SARS-CoV-2-positive patients are unclear (Zhu et al., 2020). Also, many studies in this field are case reports or case series and a limited number of narrative review articles exist in this regard (Cai et al., 2022; Flegr, 2021; Głuchowska et al., 2021; Miguel et al., 2021). There is a demanding need to combine all of the reported parasitic disease-COVID-19 co-infection cases to understand the disease's special conditions and to unlock any possible relationship between them. Therefore, the study aimed to systematically review the cases of co-infection of parasitic disease and COVID-19.

## 2 Materials and methods

### 2.1 Data sources

All relevant articles were recovered from three databases, namely PubMed, Scopus, and Web of sciences without any time restriction until 10-Feb-2023. The following keywords were used: “COVID-19”, “SARS-CoV-2”, “novel coronavirus:”, “2019-nCoV”, “Severe Acute Respiratory Syndrome-2”, “coronavirus disease- 2019”, “parasite”, “protozoa”, “helminths”, “parasitic fluke”, “parasitic worm”, “ectoparasites”, “malaria/Plasmodium”, “Leishmania/leishmaniasis”, “Entamoeba/ amoebiasis”, “Trypanosoma”, “Giardia”, “Toxoplasma”, “Lophomonas/Lophomoniasis”, “Acanthamoeba”, “Babesia”, “Balamuthia”, “Cryptosporidium”, “Cyclospora”, “Naegleria”, “Ascaris”, “Pinworm”, “Strongyloides/trongyloidiasis”, “Toxocara”, “Guinea worm/dracunculiasis”, “Hookworm”, “Tapeworm/cysticercosis”, “echinococcosis”, “Whipworm/Trichuris”, “Schistosoma”, “Gnathostoma”, “Paragonimus”, “Fasciola”, “Trichobilharzia”, “Chigoe flea/Tunga”, “maggot/myiasis”, “screwworm”, “louse”, “Tick/Ixodoidea”, “Flea”, “Mosquito”, “papular urticaria”, “Bed bug”, “Chiggers”, and “mite”, alone or in combination with other operators (“AND” and/or “OR”). Preferred Reporting Items for Systematic Reviews and Meta-Analysis (PRISMA) guidelines were complied with for conducting the study (Liberati et al., 2009).

### 2.2 Study selection

The full texts of the studies were read accurately by two independent authors and any discrepancy was resolved through discussing with other authors. The following information was retrieved from each study: authors, article type, publish/accepted date, place of study, resident or travel to parasite endemic region, parasitic disease, parasite species, study population, population number, number of COVID-19 -parasitic disease co-infected case/cases, gender, age, clinical background/risk factors, COVID-19 confirming method, parasite confirming method, disease manifestation, treatment regimen, treatment time, outcome, and any special/innovative points. A code was given to each study for easy referring.

### 2.3 Data extraction

The studies about COVID-19 co-infected with any parasitic disease (caused by protozoa, helminths, flukes, or ectoparasites) were included. Articles that have not reported COVID-19 -parasitic disease co-infected case/cases, hypothesis, conferences, books, comments, letters, and review articles without reporting any co-infected case were excluded. Also, the articles that fully published in non-English languages and even their abstract was non-English, were excluded. In addition, pre-print, unavailable full-text, ecological, vaccine, and non-human studies were excluded.

## 3 Results

### 3.1 Literature search results

**Fig. 1:**
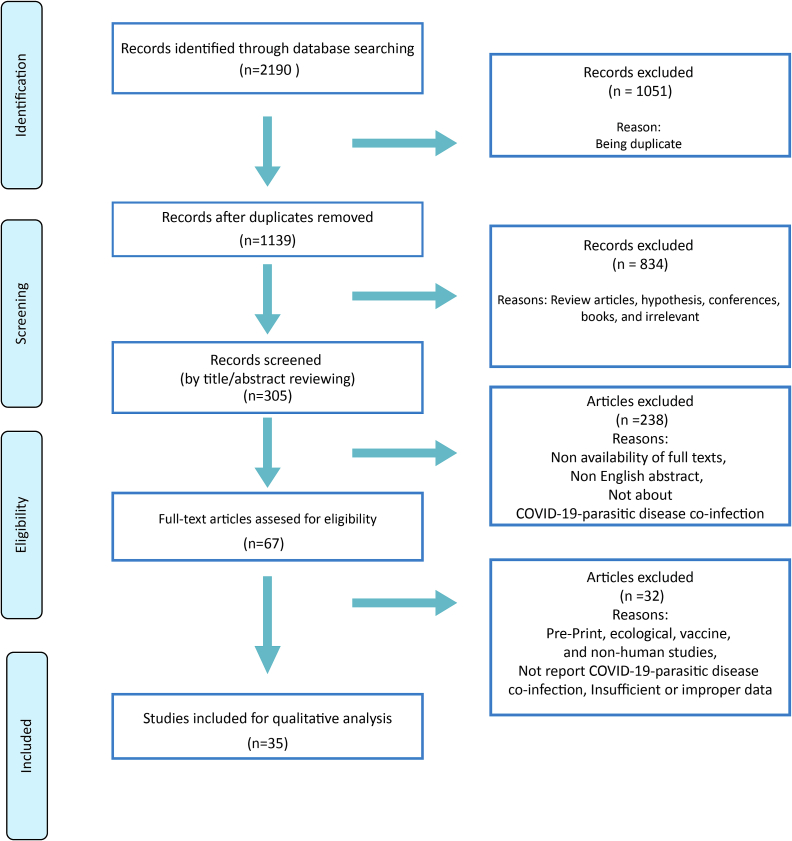
The study selection flowchart.

A total of 2190 records were recovered from databases (953 from records Pubmed, 872 records from Scopus, and 365 records from Web of Science). Of these records, 1051 records were excluded due to being duplicates. Then, 834 non-relevant studies were excluded. Of 305 remaining records, 238 were excluded through accurate title/abstract reading and 67 remained for full-text reading. After full-text reading, 32 studies were excluded which have not properly report COVID-19-parasitic disease co-infection cases and 35 studies remained for final data extraction and systematic analysis (Fig. 1). Since the obtained data were varying tremendously, meta-analysis was not conducted.

### 3.2 Records general data

The data of the final included studies are given in Table 1. The most frequent article types were case report (18 studies), followed by research article (eight studies), letter-to-editor (three studies), brief communication (two studies), case series (two study), observational (one study), and symposium (one study). The majority of studies were about COVID-19 co-infected with malaria (19 studies), followed by strongyloidiasis (three studies), amoebiasis (two studies), chagas (two studies), filariasis (one study), giardiasis (one study), leishmaniasis (one study), lophomoniasis (one study), myiasis (one study), toxoplasmosis (one study), and other various parasitic diseases (three studies). The most-reported COVID-19 co-infected parasites were *Plasmodium vivax*, *Plasmodium falciparum*, *Entamoeba histolytica*, *Strongyloides stercoralis*, *Giardia lamblia*, and *Trypanosoma cruzi*. The most co-infected cases were adult (19–59 years) men. The most frequently-used methods for COVID-19 and parasites detection were RT-PCR and microscopic methods, respectively. None of the included studies reported previous/recurrence COVID-19 infection of the cases.

**Table 1:**
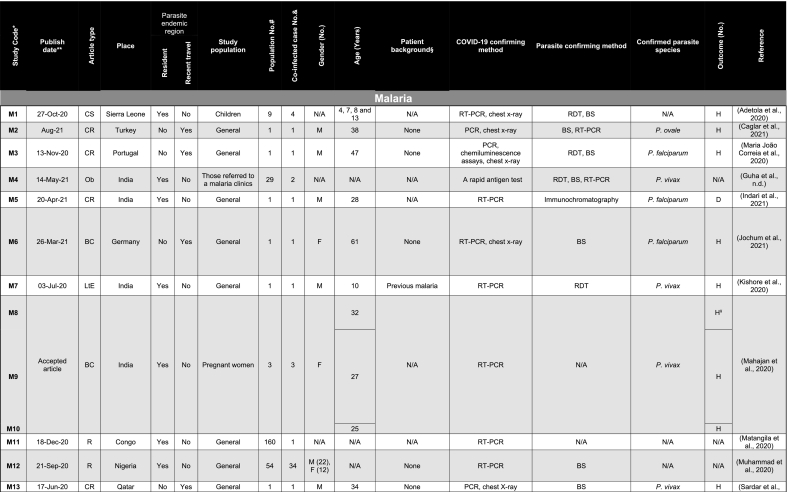

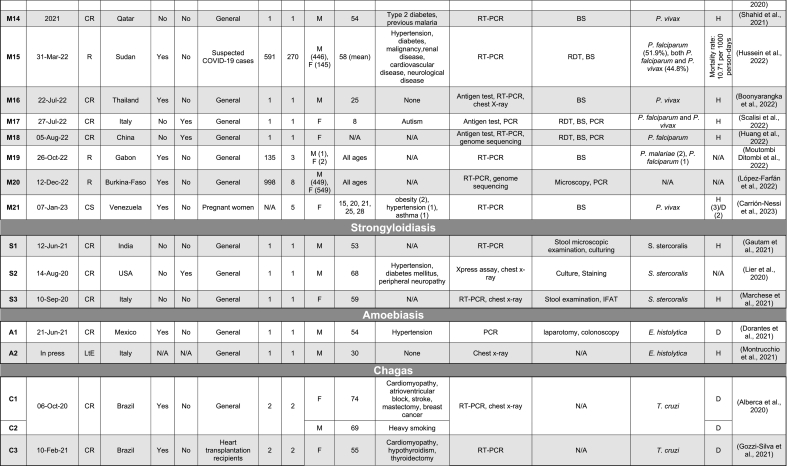

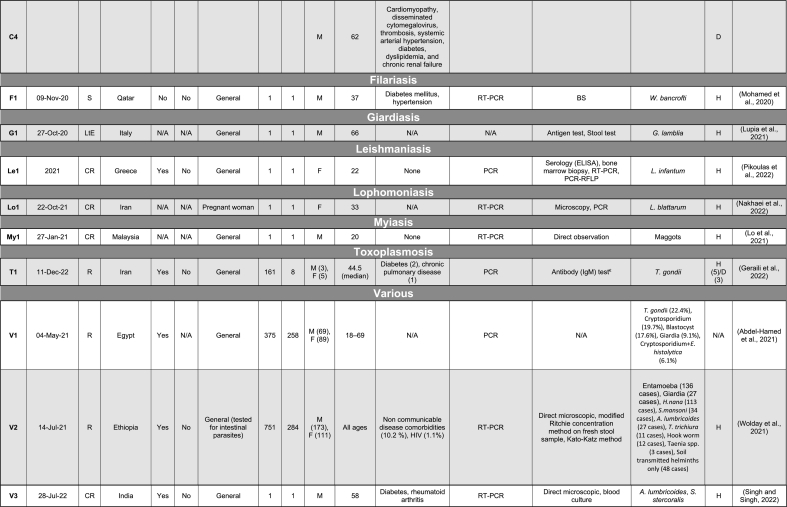
Characteristics of studies on COVID-19 and parasites co-infection (Adetola et al., 2020; Caglar et al., 2021; Correia et al., 2020; Guha et al., 2023; Indari et al., 2021; Jochum et al., 2021; Kishore et al., 2020; Mahajan et al., 2020; Matangila et al., 2020; Muhammad et al., 2020; Sardar et al., 2020; Shahid et al., 2021; Hussein et al., 2022; Boonyarangka et al., 2022; Scalisi et al., 2022; Huang et al., 2022; Moutombi Ditombi et al., 2022; López-Farfán et al., 2022; Carrión-Nessi et al., 2023; Gautam et al., 2021; Lier et al., 2020; Marchese et al., 2021; Dorantes et al., 2021; Montrucchio et al., 2021; Alberca et al., 2020; Gozzi-Silva et al., 2021; Mohamed et al., 2020; Lupia et al., 2021; Pikoulas et al., 2022; Nakhaei et al., 2022; Lo et al., 2021; Geraili et al., 2022; Abdel-Hamed et al., 2021; Wolday et al., 2021; Singh and Singh, 2022).

Abbreviations: CS: case series, CR: case report, R: research article, S: symposium, LtE: letter to editor, Ob: observational, BC: brief communication, M: male, F: female, PCR: polymerase chain reaction, RT-PCR: real time PCR, RDT: rapid diagnosis test, BS: blood smear, IFAT: immunofluorescence antibody test, *P. falciparum*: *Plasmodium falciparum*, *P. ovale*, *Plasmodium ovale*, *P. vivax*, *Plasmodium vivax*, *S. stercoralis*: *Strongyloides stercoralis*, *E. histolytica*: *Entamoeba histolytica*, *T. cruzi*: *Trypanosoma cruzi*, *W. bancrofti*: *Wuchereria bancrofti*, *G. lamblia*, *Giardia lamblia*, *L. infantum*: *Leishmania infantum*, *L. blattarum*: *Lophomonas blattarum*, *T. gondii*: *Toxoplasma gondii*, *H. nana*: *Hymenolopis nana*, *S.mansoni*: *Schistosoma mansoni*, *A. lumbricoides*: *Ascaris lumbricoides*, *T. trichiura*: *Trichuris trichiura*, H: healed and discharged treated, D: died, N/A: not available.

^⁎^ A code was used for each study (if an article described more than one case or case series, each one was specified with a unique code), These codes are concordant in codes used in Table 2.

^⁎⁎^ In some studies, the publish date was unknown so the accepted date was used.

^#^ Number of individuals studied.

^&^ Number of COVID-19 patients co-infected with parasites.

^€^ In this study, both anti-toxoplasma IgM and IgG were assayed, but we only considered IgM as an indicator of acute toxoplasmosis.

^§^ Only severe underlying diseases/risk factors of the patients are given.

^¥^ The patient (mother) healed but she had to undergo abortion.

### 3.3 Frequency of COVID-19-parasitic disease co-infection

The majority of the studies reporting COVID-19-parasitic disease co-infection were case reports or cases series that focused on one or a very low number of co-infected cases. Therefore, the evaluating of the co-infection prevalence was not applicable. Only two studies used large general populations (article codes of V1 and V2 in Table 1), which were both in parasitic endemic regions. In one study (article code of V1), the researchers focused on the Egyptian population to investigate the role of interferon (IFN)-γ in the possible connection of COVID-19 and parasitic diseases. In this study, 258 out of 375 (68.8%) were positive for COVID-19-parasitic disease co-infection. Another study (article code of V2) tested intestinal parasitic diseases co-infected with COVID-19 in the Ethiopian population to evaluate the hypothesis that co-infection with parasites may mute COVID-19 hyper-inflammation severe responses. In this study, 284 out of 751 (37.8%) were positive for COVID-19-parasitic disease co-infection. Note that there were some other studies using relatively large sample sizes but usually, each focused on specific parasites that were not easily and truly combinable (Table 1).

### 3.4 Place of COVID-19-parasitic disease co-infection

**Fig. 2:**
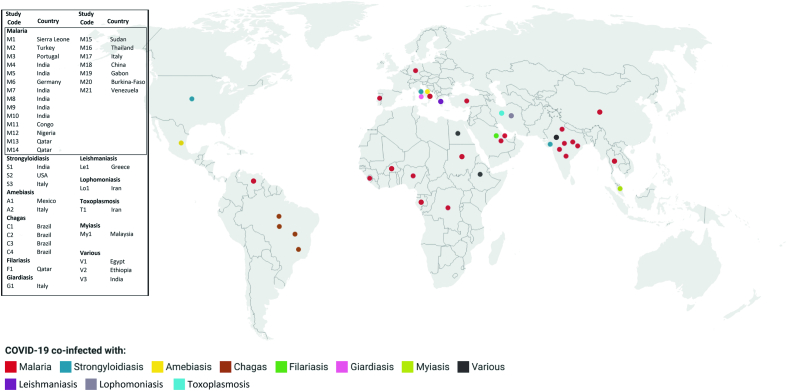
The place of reports on parasitic diseases-COVID-19 con-infection cases. The map indicates the studies on parasitic diseases-COVID-19 co-infection in different countries of the world. The map was drawn using Datawrapper server (https://www.datawrapper.de/).

The COVID-19-parasitic disease co-infection was reported in different parts of the world including India (six studies), Italy (four studies), Qatar (three studies), Brazil (two studies), Iran (two studies), Burkina-Faso, China, Congo, Egypt, Ethiopia, Gabon, Germany, Greece, Malaysia, Mexico, Nigeria, Portugal, Sierra Leone, Sudan, Thailand, Turkey, and USA (one study of each) (Fig. 2). Most cases were residents or had recently traveled to parasite endemic countries (Table 1).

### 3.5 Clinical background and manifestations of the COVID-19-parasitic disease co-infected cases

No clinical backgrounds (underlying diseases) were observed in cases of nine studies, while different clinical backgrounds were reported in the co-infected cases of 13 studies in which hypertension and diabetes were the most frequent ones. The clinical background of cases in 13 studies was not reported (Table 1).

Clinical manifestation of COVID-19-parasitic disease co-infected cases dependent on the parasitic diseases. In COVID patients co-infected with malaria, the most frequent clinical manifestations at the time of admission to hospitals/clinics were fever, abdominal pain, myalgia, chills and rigors, respiratory distress, cough, headache, and diarrhea. The most frequent manifestations on hospitalization times were low blood pressure, tachycardia, thrombocytopenia, leukocytosis, lymphopenia, hyperbilirubinemia, and elevated C-reactive protein (CRP), aspartate aminotransferase (AST), alanine transaminase (ALT), lactate dehydrogenase (LDH), ferritin, and D-dimer (Table 2).

**Table 2:** Disease manifestation and treatments of COVID-19 patients co-infected with parasites.

Study Code⁎	Manifestation (on admission)	Manifestation (physical)	Manifestation (laboratory/special)⁎⁎	Treatment #	Treatment time (days) &	Innovative point¥
Malaria						
M1	None or very low symptoms	None or very low symptoms	Diarrhea (only in one child)	Artemether-lumefantrine, haematinics and vitamin supplements	14	–
M2	Fatigue, fever (7 days)	Low BP and respiratory rate	Thrombocytopenia,high CRP, procalcitonin, AST, ALT, LDH, ferritin, D-dimer, and WBC. Low HGB	Favipiravir, enoxaparine, vitamin D, colchicine, artesunate, primaquine (after discharge)	N/A	Pandemic SARS-CoV-2 infection may mask malaria in returning travelers.
M3	Diarrhea (5 days), malaise, high fever, diaphoresi, dry cough, tachycardic	Dehydration, respiratory changes, abdominal discomfort	Mild anemia, lymphopenia, thrombocytopenia, elevated CRP and ferritin. Parasitaemia (3.1%)	Artemether, lumefantrine	14	–
M4	N/A	N/A	N/A	N/A	N/A	–
M5	Body ache, cold, fever, drowsiness (2 days), respiratory distress	N/A	leukocytosis, thrombocytopenia, lymphocytopenia, and reduced eosinophils. Elevated SALP,DBIL, TBIL, ferritin, SGGT, ALT, and AST. Altered sensorium and signs of meningoencephalitis, severe hypoxia, bradycardia	Oxygen therapy, azithromycin, vitamin B and C, pantoprazole, cephalosporin, vancomycin, doxycycline,acyclovir, levetiracetam, dexamethasone, artesunate	4	The patient developed neurological symptoms in a short time period.
M6	Fever, myalgia, diarrhea	N/A	Thrombocytopenia, high CRP	Atovaquone-proguanil	4	Severe thrombocytopenia of 23,000/μL and CRP of 10^3 mg/L were considered atypical for a clinically mild case of COVID-19
M7	Fever, chills and rigors, headache, cold, cough, abdominal pain	N/A	Thrombocytopenia	Routine malaria and COVID-19 treatments, Primaquine (after discharge)	14	They postulated that the COVID-19 may responsible for malaria relapse in the studied case
M8	Abdominal pain, headache and blurring of vision (10 days), breathing difficulty (7 days), fever with chills (3 days)	N/A	High AST and ALT	Antibiotic, labetalol, nifedipine, chloroquine	13	In cases of co-infection, the symptoms do not aggravate or present differently compared to non-co-infected COVID patients.
M9	Fever (7 days)		N/A	Antibiotic, chloroquine	15	
M10	Fever and breathing difficulty (3 days)		Leukocytosis, thrombocytopenia, high D-dimer	Antibiotic, heparin, chloroquine	25	
M11	N/A	N/A	Parasitaemia (16,900 parasites/μl)	N/A	N/A	Low prevalence of malaria and COVID-19 coinfection may be due to low prevalence of the study place or the considerable proportion of patients who received antimalarial drugs before hospitalization
M12	N/A	N/A	N/A	N/A	N/A	The results revealed significant increase of 8-iso-PGF2α and decreased alphatocopherol values among co-infected compared to COVID-19 naïve
M13	Fever (3 days), myalgia, vomiting, abdominal pain	Sinus tachycardia	leukopenia, lymphopenia, thrombocytopenia, elevated LDH, low haptoglobin, hyperbilirubemia. Elevated CRP, procalcitonin, lactic acid, ferritin and D-dimer. Parasitaemia (1.2%)	Artemether-lumefantrine, artesunate, primaquine (after discharge)	14	–
M14	Dry cough (5 days), fever, chills, rigors, profuse sweating, and lethargy	Tachycardiac, erythema.	Neutrophilic leukocytosis, thrombocytopenia, high CRP, high total bilirubin, parasitaemia (0.1%)	Artemether-lumefantrine, primaquine (after discharge)	14	COVID-19 co-infection compounds the dilemma of malaria relapse diagnosis due to overlapping symptoms.
M15	Fever, tiredness, cough, pain	N/A	N/A	Artemether-lumefantrine	21 (median)	Higher mortality rate was observed in the patients with co-infection.
M16	Fever, cough, chills, anosmia	None or very low symptoms	Anemia, thrombocytopenia, elevated alkaline phosphatase, elevated blood urea nitrogen, mild hyponatremia and hypochloremia	Primaquine, favipiravir	3	–
M17	Fever, inappetence,abdominal pain	Dehydration, eyelid oedema, hyperaemia, hypertrophy, rhonchi, mild hepatomegaly	Elevated CRP, procalcitonin, ferritin, bilirubin, LDH, and ALT, low platelet count	Ceftriaxone, atovaquone-proguanil, primaquine, IVIG, methylprednisolone, blood transfusion	N/A	The study reported the first case of multisystem inflammatory syndrome (MIS-C), SARS-CoV2, and *Plasmodium* species coinfection
M18	Fever, chills, fatigue, headache, pain, stomach cramp, and slightly nausea	Low BP and respiratory rate	Lymphopenia, increased CRP and serum amyloid A, slightly increased Ddimer, fibrinogen, and glucose	Piperaquine, Lopinavir, Ritonavir, interferon α-2b, traditional Chinese medicine, and more in second admission	N/A	–
M19	Fever	N/A	Thrombopenia (2)	Antimalarial selfmedication	N/A	Co-infected cases had a higher parasitaemia, a higher temperature,and were mostly infected with non-falciparum malaria.
M20	N/A	N/A	N/A	N/A	N/A	–
M21	Dry cough (4/5), fever (3/5), chills (3/5), and headache (2/5)	Dyspnea (1/5), arthralgia (1/5), and vomiting (1/5)	Mild elevations of hepatic enzymes, creatinine, and urea serum levels, mild alterations in the number of platelets, leucocytes, and neutrophils. Only one had severe anemia.	Steroids, supplemental oxygen, thrombosis prophylaxis, anti-malarial treatment	0 to 17	The study documented a high proportion of adverse outcomes (for both mother and fetus) among pregnant women with malaria-COVID-19 co-infection.

Strongyloidiasis						
S1	Fever and diarrhea (4 days), abdominal discomfort after meals (1.5 month)	N/A	Hiatus hernia, duodenal ulcer, normocytic anemia, neutrophilic leukocytosis, positive faecal occult blood test, moderate pleural effusion, interlobular septal thickening with linear fibrotic bands in bilateral lung parenchyma	Methylprednisolone, amoxicillin, clarithromycin, pantoprazole along, albendazole, ivermectin	14	Corticosteroids in COVID-19 pandemic have the potential to unearth hidden burden of strongyloidiasis.
S2	Chills, myalgia, headache, cough, nausea, worsening dyspnea	Dry mucous membranes and decreased air entry with bibasilar crackles	High CRP, ferritin, and D-dimer. Bilateral patchy airspace opacities. Bacteriemia *(Streptococcus constellatus, Citrobacter freundii, Pseudomonas aeruginosa and Staphylococcus aureus*).	Hydroxychloroquine, tocilizumab, methylprednisolone (discontinued after positive bacterial culture), ciprofloxacin, cefazolin, metronidazole, vancomycin, ivermectin and discontinuation of antibiotics (after strongyloidiasis disgnosis), albendazole, piperacillin– tazobactam	38	Screening for Strongyloides infection should be pursued in individuals with COVID-19 who originate from endemic regions before initiating immunosuppressive therapy.
S3	Malaise, nausea, vomiting, fever (7 days)	Low pO2 (57%), severe hypoxia, atrial fibrillation	Increased eosinophils	Oxygen therapy, hydroxychloroquine, lopinavir/ritonavir, dexamethasone, enoxaparin, tocilizumab, amiodarone, insulin-based treatment, ivermectin	29	Clinicians should be aware of the risk of strongyloidiasis as a complication of the treatment for severe COVID-19.

Amoebiasis						
A1	Dyspnea, anosmia, dysgeusia, severe abdominal pain	low pO2 (62%), low BP	Hepatic steatosis, bilateral pneumonia, hematochezia and a reduction in hemoglobin, mural engrossment, hypercoagulable state and signs of septic shock and neurological deterioration. Wound dehiscence with erythematous markings and edema.	Paracetamol, dexamethasone, oxygen therapy, enoxaparin, baricitnab and methylprednisolone, blood transfusion, imipenem	28	–
A2	Respiratory distress	N/A	Liver abscess	Dexamethasone, heparin, metronidazole, paromomycin	N/A	–

Chagas						
C1	Dyspnea, fever, myalgia	Low pO2, atrial fibrillation with dilatation of the pulmonary artery trunk	Thrombocytopenia, increased neutrophils, monocytes, creatinine, CRP, and urea. Blood glucose alterations, high prothrombin time, altered sodium, potassium, and magnesium in serum	Ceftriaxone,methylprednisolone, azithromycin, warfarin	17	–
C2	Respiratory distress	Low pO2	Increased blood troponin T, high levels of platelet and leukocytes, low lymphocyte counts, high CRP, high prothrombin time, increased magnesium serum, blood glucose alterations	Azithromycin, ceftriaxone, enoxaparin, methylprednisolone,piperacillin/tazobactam, vancomycin, meropenem	13	
C3	N/A	N/A	ARDS due to COVID–19; heart transplant rejection, disseminated cytomegalovirus; aggravated chronic kidney disease and pressure ulcer	Methylprednisolone, azathioprine, anti-thymocyte globulin, cyclosporine, tacrolimus, meropenem, linezolid, micafungin, vancomycin, polymyxin B, tigecycline, amikacin, fluconazole, ganciclovir, hydrocortisone	47	They reported two cases of heart transplantation recipients with concomitant infections by SARS-CoV-2, *T. cruzi*, and cytomegalovirus dissemination
C4			ARDS due to COVID–19, disseminated cytomegalovirus and pancytopenia due to hemophagocytosis	Meropenem, colistin, linezolid fluconazole, amikacin, cyclosporine, azathioprine, prednisone	21	

Filariasis						
F1	Fever (10 days), dyspnea, sore throat, cough, nausea, vomiting, diarrhea	Bilateral lung basal crackles	A mild CRP rise, faint hazy bilateral infiltrates	Hydroxychloroquine, azithromycin, diethylcarbamazine, doxycycline	N/A	–

Giardiasis						
G1	Respiratory distress	N/A	High eosinophils, lymphopenia	Metronidazole	42	The risk of giardiasis reactivation in COVID-19 patients should be considered

Leishmaniasis						
Le1	Diarrhea, fever	Tender peripheral lymphadenopathy, moderate hepatosplenomegaly	Pancytopenia, high AST and ALT, giant platelets, monocytosis	Liposomal amphotericin B, dexamethasone, enoxaparin, Remdesivir	13	–

Lophomoniasis						
Lo1	Hypertension, pain, fever, cough, shortness of breath, conjunctivitis	Bilateral moultilobar patchy ground glass opacities, alveolar consolidations, and mild to moderate pleural effusion	Anemia, leukocytosis, neutrophilia, lymphopenia, elevated ESR, CRP, and LDH	Metronidazole, enoxaparin sodium, ticlopidine, remdesivir, amikacin, colomycin, methylprednisolone pulse therapy, magnesium sulfate and N-Acetylcystein	42	The first reoport of co-infection of L. *blattarum* and COVID-19

Myiasis						
My1	Wound maggot infestation (2 days), low-grade fever	N/A	Elevated total white cell count	Removal of infected tissues and wound debridement	9	–


Toxoplasmosis						
T1	Fever (1), cough (3), shortness of breath (5)	N/A	N/A	N/A	N/A	No significant relationship were observed between toxoplasmosis and the symptoms of COVID-19

Various						
V1	N/A	Chest manifestation either alone (54.7%) or in association with gastrointestinal manifestations (19.7%)	COVID-19 mild (92.3%), or severe (7.7%)	N/A	N/A	The remarkable adaptation of human immune response to COVID-19 infection by parasitic infections with high levels of IFN-γ was observed in moderate cases compared with low levels in extreme cases
V2	N/A	More frequent symptoms include cough (28.2%), fever (16.9%), and head ache (14.8%)	COVID-19 mild (90.5%), or severe (9.5%)	Routine parasite and COVID-19 treatments	N/A	The findings may confirm the hypothesis that co-infection with parasites mutes hyper-inflammation associated with severe COVID-19.
V3	Breathlessness, dyspnea and abdominal pain, fever, sore throat	Icterus and tender hepatomegaly	Raised CRP, procalcitonin, amylase, Eosinophil count	Methylprednisolone, amphotericin B, aztreonam, hydroxychloroquine, favipiravir/remedesvir, azithromycin, ivermectin, albendazole	22	–

Abbreviations: BP: Blood pressure, pO2: partial pressure of oxygen, CRP: C-reactive protein, AST: aspartate aminotransferase, ALT: alanine transaminase, LDH: lactate dehydrogenase, WBC: white blood cell, HGB: hemoglobin, 8-iso-PGF2α: 8-isoprostaglandin F2α, SALP: serum alkaline phosphatases, DBIL: bilirubin-direct, TBIL: bilirubin total, SGGT: serum gamma-glutamyl transferase, ARDS: acute respitory distress syndrom, N/A: not available.

⁎ A code was used for each study (if an article described more than one case or case series, each one was specified with a unique code). These codes are concordant in codes used in Table 1 so their references could be find in Table 1.

⁎⁎ Only those parameters that were outside of the normal ranges are given. Parasitaemia have been reported only in some studies, although all have confirmed the parasite diseases in their studied cases.

# Only the main drug used are given. In all studies the routine COVID-19 have been applied, although only some of them declared the details.

& The time after the patients diagnosis (admission) until outcome (discharge or death) are given.

¥ Only some important and innovative points of studies are given.

In COVID patients co-infected with strongyloidiasis the most common manifestations were fever, low partial pressure of oxygen (pO2), patchy airspace opacities, leukocytosis, and elevated eosinophils, CRP, ferritin, and D-dimer (Table 2).

The most frequent manifestations in chagas-COVID-19 co-infected cases were increased blood troponin T, blood glucose alteration, low pO2, leukocytosis, and elevated CRP (Table 2). In two cases of COVID-19 co-infected with amoebiasis, the liver abscess was observed in addition to general symptoms of COVID-19. The clinical manifestations of COVID-19 cases co-infected with other parasites were as reported in Table 2.

### 3.6 Treatment and outcome of the COVID-19-parasitic disease co-infected cases

The majority of the COVID-19-parasitic disease co-infected cases were healed under appropriate therapeutic management (Table 1). The treatment regimens of the COVID-19-parasitic disease co-infected cases were varying and dependent on the severity of COVID-19 and the co-infected parasite. In all cases, routine therapeutic managements were applied, although only some studies mentioned its details. The most anti-COVID-19 drugs used were oxygen therapy, lopinavir/ritonavir, favipiravir, enoxaparin, hydroxychloroquine, dexamethasone, tocilizumab, heparin, and vitamins supplements. The most frequently used anti-parasite drugs for COVID-19 patients co-infected with malaria, strongyloidiasis, and chagas were artemether-lumefantrine, ivermectin, and azithromycin, respectively (Table 2).

## 4 Discussion

In the present study, we tried to recover published studies of COVID-19 co-infected with almost all parasitic diseases. However, for many parasites, no co-infection report existed. Given the existence of chronic parasitic diseases in the world, and with the sheer millions of COVID-19 infections, the number of co-existing infections would be staggering but not reported. Previous studies have shown that some parasitic diseases such as schistosomiasis, malaria, and helminths, may increase the risk of severe COVID-19 infection (Cai et al., 2022; Głuchowska et al., 2021). However, as our systematic review showed, even for those parasites that co-infection with COVID-19 has been reported the number of studies is very low (mainly one or two studies). This issue indicates that co-infection of parasites and COVID-19 is not frequently reported. Also, due to the low number of studies and low sample size of many of them, calculating the prevalence of COVID-19-parasitic diseases co-infection was not applicable.

In comparison to other parasitic diseases, more studies exist on malaria-COVID-19 co-infection. There are some possible reasons for this as follows: 1- Malaria is widely spread around the world (Garcia, 2010), 2- It seems that there is a low prevalence of COVID-19 in malaria-endemic countries (Anyanwu, 2021). The difference in COVID-19 prevalence between malaria-endemic and non-endemic countries may be attributed to several factors like mitigation tools adopted, testing capacity, or cultural habits, although much more theories have been given that are summarized elsewhere (Hussein et al., 2020). 3-In the first months after COVID-19 pandemic, some anti-malaria drugs such as hydroxychloroquine are reported to be effective against COVID-19 (Prodromos and Rumschlag, 2020), 4- It has been declared that malaria and COVID-19 have some common mechanisms of pathogenicity (Di Gennaro et al., 2020), 5- Human immune responses against malaria and COVID-19 are reported to have some similarities (Di Gennaro et al., 2020). These issues attract researchers to conduct more studies on the malaria-COVID-19 relationship. However, the majority of them focused on the possible common mechanism of these two diseases and attempted to find any effective dug against COVID-19, resulting in a relatively lower number of reports describing malaria-COVID-19 co-infection cases.

The most frequent COVID-19-parasitic diseases co-infection was observed in adult men. It may be due to the fact that men have greater access to healthcare versus women and may be overrepresented because of this. Also, the parasitic infections are underreported in women due to several barriers to care such as higher levels of poverty, lower education and social status (Wharton-Smith et al., 2019).

The co-infection was reported in different parts of the world but mainly in those endemics for the parasites. Even the most reporting cases in non-endemic countries had recently traveled to the parasites' endemic regions. Therefore, it is obvious that the probability of COVID-19-parasitic diseases co-infection is higher in parasites' endemic regions. Note that in the literature, other studies than our included articles might also discuss parasite-COVID-19 co-infection, but they were excluded for reasons in the present study. Therefore, the countries presented here are those included in our systematic review and not exclusively the definite countries where co-infected cases have been observed.

Some parasites such as *S. stercoralis* are opportunistic parasites that may latently live in human bodies and appear after the weakening of the immune system. Since corticosteroids which are immune response suppressants are used to treat COVID-19, latent parasites have the opportunity to emerge and cause disease (Gautam et al., 2021). Therefore, it has been suggested that before initiating immunosuppressive therapy, screening for opportunistic parasites such as *S. stercoralis* be pursued in COVID-19 patients who originate from endemic regions (Gautam et al., 2021; Lier et al., 2020; Marchese et al., 2021). Furthermore, COVID-19 disease condition and its treatment regimens may cause re-emergence of some previous parasitic diseases as seen for malaria relapse in two co-infected cases (Kishore et al., 2020; Shahid et al., 2021), and giardiasis reactivation in one case (Lupia et al., 2021).

Leishmaniasis is an NTD that has different manifestations from the self-limiting cutaneous type to the fatal visceral type and is caused by several Leishmania species (Maxfield and Crane, 2022; Rostamian and Niknam, 2019). Because cutaneous leishmaniasis has a low life risk, it is neglected, especially in the COVID-19 pandemic era. For this reason, it seems co-infected cases of cutaneous leishmaniasis and COVID-19 may not be sent for publication in scientific journals. Therefore, it is likely that cutaneous leishmaniasis-COVID-19 co-infection is much more common than reported in the current study. On the contrary, its visceral leishmaniasis is clinically valuable due to the possibility of lethality, and for this reason, its co-infection with COVID-19 is more worth reporting. However, there are limited, but increasing cases of this type of co-infection in the information sources (Colomba et al., 2022; Paul and Singh, 2023), although this type has a wide range in the world from Southeast Asia to the Middle East, Africa, and South America (Kone et al., 2019; Rostamian et al., 2021; Scarpini et al., 2022). As with other neglected parasites, the co-infection of Leishmania with COVID-19 needs further investigation.

Although hypertension and diabetes were the most frequent underlying diseases observed in the co-infected cases and could be assumed as risk factors, the number of reports is low and more studies are needed to confirm it. It is noteworthy that hypertension and diabetes are two main risk factors of COVID-19 severity (de Almeida-Pititto et al., 2020). Therefore, it seems logical that co-infection with parasites, parallel to COVID-19 severity, be more prevalent in individuals with hypertension and diabetes.

Clinical manifestation of COVID-19-parasitic disease co-infected cases dependent on the parasitic diseases. These manifestations seem to be no different from routine COVID-19 or parasitic diseases, separately, although only some studies mentioned this issue (Mahajan et al., 2020). However, two studies on malaria-COVID-19 co-infection showed a somewhat different manifestation of co-infected cases in comparison to COVID-19 naive. In one study, severe thrombocytopenia (23,000/μL) and CRP of 1000 mg/L were observed in the co-infected cases that were considered atypical for a clinically mild case of COVID-19 (Jochum et al., 2021). Another study reported decreased alpha-tocopherol values and a significant increase of 8-isoprostaglandin F2α (8-isoPGF2α) among co-infected cases compared to COVID-19 naïve (Muhammad et al., 2020). It is also noteworthy that due to some overlapping symptoms and more frequent cases of COVID-19 in the pandemic era, most co-infected cases have been initially diagnosed as COVID-19 and later the parasitic infection has been found as an incidental finding.

The standard common treatments for COVID-19 and parasitic diseases seem to be effective since the majority of co-infected cases healed and only those who were old and had severe forms of COVID-19 died. The challenging issue for the treatment of co-infected patients is their accurate and on-time diagnosis because COVID-19 and some parasitic diseases such as malaria have many overlapping symptoms that make their diagnosis difficult (Di Gennaro et al., 2020; Shahid et al., 2021).

Altogether, here we compiled reporting cases of parasitic diseases-COVID-19 co-infections and found out that: 1- There are a relatively lower number of reports on parasitic diseases-COVID-19 co-infections in the world compared to co-infection with other microorganisms, 2- The most co-infected cases are adult men who resident of or travel to parasite-endemic countries, 3- No or low manifestation differences exist between the co-infected cases and naïve COVID-19 or naïve parasitic disease, 4- COVID-19 conditions and treatment regimens may cause parasites re-emergence, relapse, and re-activation, 5- If the parasitic disease and COVID-19 diagnosed accurately and on-time, the patients will be treated faster and more efficiently. It should be noted that since diagnostic strategies for acute COVID-19 infection are considerably variable and diagnostic test positivity does not always confirm active infection (Vandenberg et al., 2020), the meaning of co-infection should be considered carefully. We also suggest that more accurate studies on general populations of endemic regions with larger sample sizes be conducted to find any relationship between parasitic diseases and COVID-19.

The present study faces some limitations as follows: 1- We excluded reviews, comments, letters, and conferences, consequently the data gathered here may not represent all the existing data in the field, 2- The cases that had the latent type of parasitic diseases (such as latent toxoplasmosis) and were later infected with COVID-19 were excluded from the study, and 3- An article on Chagas-COVID-19 co-infection (Molina et al., 2021), was excluded because it selected the co-infected cases and compared them with non-co-infected cases. Since this strategy does not show the real number of co-infected cases it was excluded, however, its data may be also valuable in this case.

## 5 Conclusion

Although there was a relatively low number of reports on parasitic diseases-COVID-19 co-infection, COVID-19 and some parasitic diseases have overlapping symptoms and also COVID-19 conditions and treatment regimens may cause some parasites re-emergence, relapse, or re-activation. Therefore, more attention should be paid to the on-time diagnosis of COVID-19 and the co-infected parasites.

## Authors' contributions

MR and KGH conceived the study; MR designed the study protocol; FN analysis and interpretation of these data. FN and SK drafted the manuscript; MR and HM critically revised the manuscript for intellectual content. All authors read and approved the final manuscript.

## Funding

This study is supported by 10.13039/501100005317Kermanshah University of Medical Sciences.

## Declarations of Competing Interest

None.
